# Shared decision making in surgery: A scoping review of the literature

**DOI:** 10.1111/hex.13105

**Published:** 2020-07-22

**Authors:** Kacper Niburski, Elena Guadagno, Sadaf Mohtashami, Dan Poenaru

**Affiliations:** ^1^ McGill Medicine McGill University Montreal QC Canada; ^2^ Division of Pediatric General and Thoracic Surgery The Montreal Children’s Hospital McGill University Health Centre Montreal QC Canada

**Keywords:** patient‐centred care, shared decision making, surgery

## Abstract

**Background:**

Shared decision making (SDM) has been increasingly implemented to improve health‐care outcomes. Despite the mixed efficacy of SDM to provide better patient‐guided care, its use in surgery has not been studied. The aim of this study was to systematically review SDM application in surgery.

**Design:**

The search strategy, developed with a medical librarian, included nine databases from inception until June 2019. After a 2‐person title and abstract screen, full‐text publications were analysed. Data collected included author, year, surgical discipline, location, study duration, type of decision aid, survey methodology and variable outcomes. Quantitative and qualitative cross‐sectional studies, as well as RCTs, were included.

**Results:**

A total of 6060 studies were retrieved. A total of 148 were included in the final review. The majority of the studies were in plastic surgery, followed by general surgery and orthopaedics. The use of SDM decreased surgical intervention rate (12 of 22), decisional conflict (25 of 29), and decisional regret (5 of 5), and increased decisional satisfaction (17 of 21), knowledge (33 of 35), SDM preference (13 of 16), and physician trust (4 of 6). Time increase per patient encounter was inconclusive. Cross‐sectional studies showed that patients prefer shared treatment and surgical treatment varied less. The results of SDM per type of decision aid vary in terms of their outcome.

**Conclusion:**

SDM in surgery decreases decisional conflict, anxiety and surgical intervention rates, while increasing knowledge retained decisional satisfaction, quality and physician trust. Surgical patients also appear to prefer SDM paradigms. SDM appears beneficial in surgery and therefore worth promoting and expanding in use.

## 1 INTRODUCTION

Shared decision making (SDM) is a collaborative model of care that allows patients and clinicians to mutually agree on treatment based on their contextual values and preferences. Underlying it is the conceptual framework of internalizing, and realizing, a patient's system of decision making.^1^ This model has been found to prevent indiscriminate medical interventions, provide a means for patients to determine value‐based health outcomes particularly when multiple options exist, and prevent variation in care paradigms across hospitals.^2^, ^3^, ^4^ These outcomes have made SDM an essential component of patient‐centred care.

Frameworks exist for clarifying the implementation of an SDM platform, such as the Makoul Model.^5^ These conceptual foundations are operationalized in evidence‐based decisional aids (DAs). Increasingly, such DAs are used to promote SDM in clinical settings, as well as increase patient engagement in health‐care settings. Numerous randomized controlled trials (RCTs) have shown that DAs improve the quality of decision making, increase the knowledge received and retained from clinicians, and decrease decisional conflict.^6^, ^7^, ^8^

Despite the proven efficacy of SDM to provide better patient‐guided care in medicine, its use in surgery has not been widely studied. Historically, this has largely been due to a focus on informed consent as a means to save surgical time.^9^ For example, decision making in surgery is often considered one of equipoise: no one option exists, unless life‐saving measures are required.

A recent Cochrane review has shown similar results. It included only 15 surgical studies, the majority of which were in plastic surgery.^10^ Another systematic review only examined elective surgeries.^11^ This paucity of studies may be due to a perceived need to concentrate on information‐giving in surgical encounters, as opposed to having the time to discuss alternative treatments, recognize patients’ beliefs, and determine if the associated risks and benefits are understood.^12^ Yet unlike other disciplines, surgery is often irreversible, potentially resulting in a radical change in life and health status. This permanency of surgery would greatly benefit from SDM, especially when weighing the ultimate long‐term consequences of surgical solutions vs alternative, non‐interventional therapies.

The aim of this study is to systematically review SDM in surgery. This includes investigating the SDM impact on patients’ decisional conflict, knowledge, and time spent in surgical consultation, its use in surgical subspecialties, its use in subsets of surgical populations such as minorities, and surgical patients’ perceptions of SDM in their care. The study further seeks to qualitatively compare the efficacy of surgical decisional aids in their surgical disciplines.

## 2 METHODS

The protocol for the study was designed in concordance with the PRISMA (Preferred Reporting Items for Systematic Reviews and Meta‐Analyses) checklist and registered with PROSPERO (CRD42018097286), the international registry for systematic reviews.^13^, ^14^ The search strategy was conducted by a senior medical librarian. The following databases were searched from the inception of the databases 7 June 2018 and updated 25 June 2019: Medline (Ovid), Embase (Ovid), Cochrane (Wiley), Africa‐Wide (Ebsco), Global Health (Ovid), Global Index Medicus (WHO), and Web of Science (Clarivate Analytics), with no language restrictions. A separate grey literature search was conducted, including the WHO Emergency and Essential Surgical Care (EESC) global database. The search strategy used variations in text words found in the title, abstract or keyword fields, and relevant subject headings to retrieve articles pertaining to SDM in surgery. The concepts of shared decision making, decisional aids and support techniques were combined with variations of patient satisfaction or patient‐centred care combined with surgical terms (see the full search strategy for details).

Necessary to the search was an operationalized definition of SDM, as occasionally the term has been used incorrectly.^15^ The following working definition was determined most appropriate: ‘an approach where clinicians and patients share the best available evidence when faced with the task of making decisions, and where patients are supported to consider options and to achieve informed preferences’.^16^ Such a definition was used by^1^ in the most comprehensive SDM review of medical specialties.^16^ Moreover, it is fitting for surgery, where best evidence may require other paradigms besides SDM to be used, such as in the case of emergency surgery.^17^ Given the definition, competing SDM frameworks were not included in the search. This resulted in some papers being excluded based on other forms of patient–physician relationship, such as informed consent only.^18^

### 2.1 Inclusion and exclusion criteria

Publications were included if they discussed SDM by surgical discipline. Both emergency and elective procedures were included. Studies were excluded if they did not contain explicit methods for quantifying SDM; if they were commentaries, opinion pieces, reviews, committee reports, or conference abstracts; and if surgery was not made the explicit outcome in the abstract. Studies were also excluded if they discussed other means of shared patient information, such as genetic screening. This resulted in including both qualitative and quantitative cross‐sectional studies, as well as randomized controlled trials.

**FIGURE 1:**
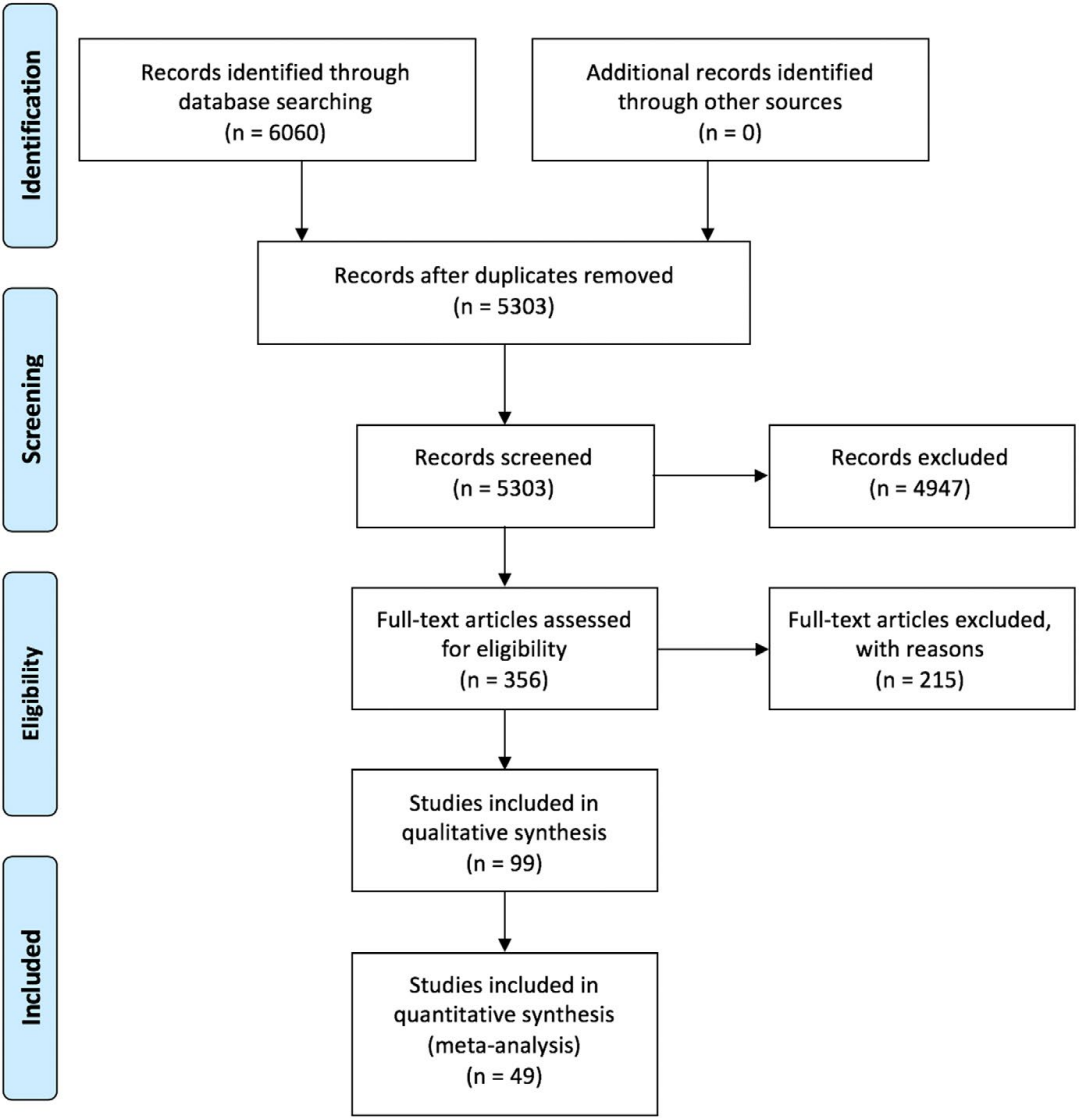
Identification of eligible studies in scoping review

Two investigators (KN and SM) independently reviewed all eligible titles and abstracts. In instances of disagreement, a third reviewer (DP) adjudicated. A full‐text screen was then performed to determine acceptability into the final review (Figure 1). This was done by KN to ensure inclusion criteria were met, if the studies used SDM in a surgical discipline only, and to understand how the studies differed in their use of SDM.

### 2.2 Data extraction and quality assessment

Data were extracted from two types of publications: controlled comparative studies and exploratory cross‐sectional studies. For each, the author(s), year, surgical discipline, total number of patients, number of sites, location, median patient age, study duration, whether a decisional aid was used, intervention type, survey methodology and variable outcomes were recorded (Table 1). For the controlled comparative studies, the total number of participants in controls compared to the intervention group was recorded. For the cross‐sectional studies, additional qualitative comments and themes were noted. This included two authors (KN and EG) to categorize the papers individually. The two identified, coded and grouped relevant text using Excel ^(4)^. The common words were grouped by frequency and similarity *post hoc,* in a method similar to the Joanna Briggs Institute guidance.^19^

**TABLE 1:** Study data (n = 140)

Study design	
Total	148
RCT	49
Cross‐sectional	99
Size	
Single	100
Multicentre	48
Sample size	
<100	70
100‐500	69
>500	9
Population age	
No age	53
<18	3
18‐50	23
50‐65	56
>65	13
Continent	
North America	102
Europe	30
Asia	10
Other	6
Surgical specialty	
Neurosurgery	2
Orthopaedic	27
Urology	15
Obstetrics	11
General Surgery	18
Plastics	39
Otolaryngology	8
Cardiovascular	8
Transplant	9
Vascular	4
Gastrointestinal	7

The quality of each RCT was analysed manually using the Cochrane Bias Risk Assessment tool (Table 2). RCTs were additionally grouped based on the type of decisional aid used, the study design type (preoperative or postoperative), and the surgical speciality. The observational studies were not coded using an assessment tool due to their heterogeneity.

**TABLE 2:** Cochrane bias risk assessment tool findings of RCTs

	Selection bias		Blinding		Other biases	
	Random sequence	Allocation concealment	Performance bias	Detection bias	Attrition bias	Reporting bias
Juraskova et al. (2015)	Low	Low	Low	Low	Low	Unknown
Brazeli et al. (2015)	Low	Unknown	High	High	Unknown	Unknown
Stalmeier et al. (2009)	Low	Low	Low	High	Low	Unknown
Tucholka et al. (2017)	Low	Low	Low	High	Low	Unknown
Trenaman et al. (2017)	Low	Low	Low	High	Low	Unknown
Shirk et al. (2017)	Low	High	High	High	Low	Unknown
Korteland et al. (2017)	Low	Low	High	High	Low	Unknown
Ibrahim et al. (2017)	Low	Low	High	High	Low	Unknown
Basu et al. (2016)	Low	Low	High	High	Low	Unknown
Warner et al. (2015)	Low	Low	High	High	Low	Unknown
Sawka et al. (2015)	Low	Low	Low	High	Low	Unknown
LeBlanc et al. (2015)	Low	Low	Low	Low	Low	Low
Fraval et al. (2015)	Low	Low	High	High	Low	Unknown
Knops et al. (2015)	Low	Low	High	High	Low	Unknown
Ibrahim et al. (2013)	Low	Low	Low	Low	Low	Unknown
Schwaim et al (2012)	Low	Unknown	High	High	Low	Unknown
Vodermaier et al. (2011)	Low	Unknown	High	Low	Low	Unknown
Vandemheen et al. (2009)	Low	High	High	High	Unknown	Unknown
Raynes‐Greenow et al. (2009)	Low	Low	High	High	Low	Unknown
Wong et al, (2006)	Low	Low	High	High	Low	Unknown
Jibaja‐Weiss et al. (2006)	Low	Low	High	High	Low	Low
Whelan et al. (2004)	Low	Low	High	High	Low	Unknown
Vuorma et al. (2003)	Low	Low	High	High	Low	Unknown
Kenney et al. (2002)	Low	Unknown	High	High	Low	Unknown
Goel et al. (2001)	Low	Low	High	Low	Low	Unknown
Brito et al. (2015)	Low	Low	High	High	Low	Unknown
de Achaval et al. (2012)	Low	Low	High	High	Low	Unknown
Hoffman et al. (2014)	Low	Low	High	Low	Low	Unknown
Serpico et al. (2016)	Low	Low	High	High	Low	Unknown
Rolving et al. (2015)	Low	Low	High	High	Unknown	Unknown
Causarano et al. (2015)	Low	Low	High	Unknown	Low	Unknown
Van Tol‐Geerdink et al. (2016)	Low	High	High	High	Low	Low
Patzer et al. (2018)	Low	Low	High	High	Low	Unknown
Osaka et al. (2017)	Low	Low	Low	Low	Low	Unknown
Berger‐Hogerip et al. (2017)	Low	Low	High	High	Low	Unknown
Stacey et al. (2016)	Low	Low	Low	High	Low	Unknown
Shue et al. (2016)	Low	Low	Low	High	Low	Unknown
Luan et al. (2016)	Low	Low	High	Unknown	Low	Unknown
Kearing et al, 2016	Low	Low	High	Unknown	Low	Unknown
Barbers et al. (2016)	Low	Low	High	High	Low	Unknown
Stacey et al. (2014)	Low	Low	Low	Unknown	Low	Low
Van Tol‐Geerdink et al. (2013)	Low	Low	High	High	Low	Unknown
Bozic et al. (2013)	Low	Low	High	High	Low	Unknown
Vodermaier et al. (2009)	Low	Low	High	High	Low	Unknown
Lin et al. (2019)	Low	Low	Low	Low	Low	Unknown
Kostick et al. (2019)	Low	Low	High	Unknown	Low	Low
Cuypers et al. (2019)	Low	Unknown	Low	High	Low	Low
McCars et al. (2019)	Low	Low	Low	Low	Low	Unknown
Klaassen et al. (2019)	Low	Low	High	Unknown	Low	Low
Doll et al. (2019	Low	Low	Low	Low	Low	Low

## 3 RESULTS

**FIGURE 2:**
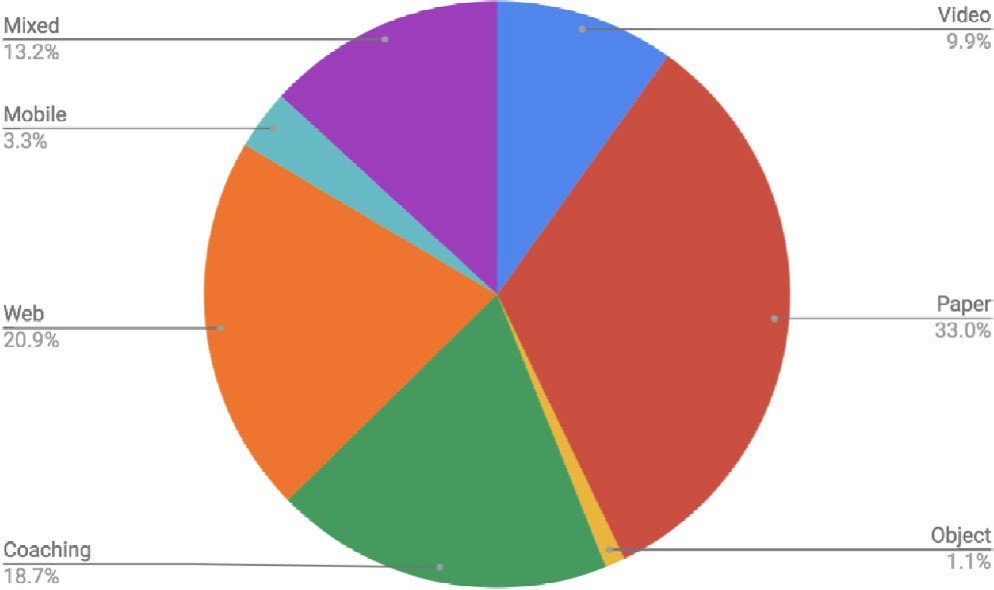
The seven types of decisional aids used to apply shared decision making

A total of 6060 studies were retrieved from the literature search, after initially searching until 7 June 2018 and then updating with 25 June 2019 papers (Figure 1). After duplicates were removed, 5303 studies were available for screening. After reviewing titles and abstracts as well as removing any additional duplicates, 356 publications were identified for full‐text review and 148 included in the final set. Table 1 illustrates the general characteristics of the final studies. The majority of the publications (67%) were single‐institution studies, with most (67%) being cross‐sectional. Forty‐eight per cent of the studies had a small sample size (less than 100 patients), 37% focused on patients aged 50‐65 years, and 69% originated in North America. The most represented specialty was plastic surgery, particularly focusing on the choice between mastectomy and non‐surgical therapy for breast cancer (Figure 2). This was followed by general surgery (for elective surgeries) and orthopaedics (primarily osteoarthritis), paediatric surgery with 3 studies, and neurosurgery with only one qualitative cross‐sectional study.

The publications selected included 99 qualitative and quantitative cross‐sectional studies and 49 RCTs. Decision aids were used in 91 RCTs and cross‐sectional studies, with the others using SDM models to elicit patient preferences. Seven types of decisional aids were employed: paper/booklet format (34.5%), web‐based (20.2%), coaching (16.7%) and mixed media that used more than one type of decisional aid (13.1%), including 80% video with paper, 10.7% video alone, 5% with a multicomponent of coaching and a paper, 3.6% mobile app, and 1.2% object‐based.

**FIGURE 3:**
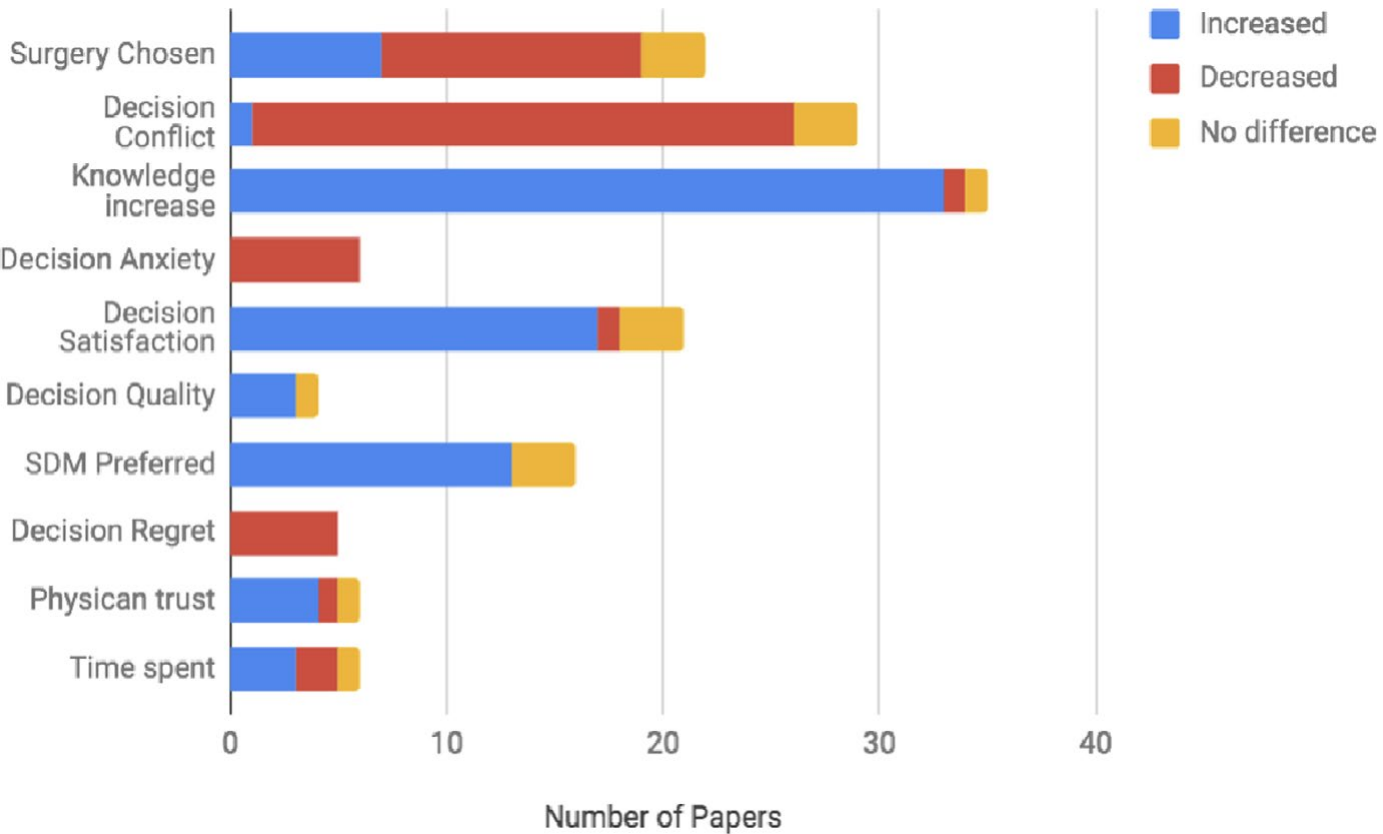
Trends of DA‐specific measures. Blue marks increase, red decrease, and yellow no change

Two themes emerged from the review: measurable outcomes from the surgical DAs, and qualitative descriptions of SDM from cross‐sectional studies. The primary outcomes measured using surgical DAs were intervention rates following SDM, decisional conflict in comparative groups, and the time required using an SDM paradigm.^20^ Secondary outcomes including decisional satisfaction, decisional regret, knowledge provided, decisional anxiety and quality, and increased patient–physician trust were also aggregated according to their definitions in the literature.^21^ These selected outcomes are the most common outcomes underpinning proper SDM paradigms.^22^ Figure 3 summarizes the specific characteristics of the DAs. The majority of the studies were of good quality, although detection and performance biases were a concern.

### 3.1 Surgical rates & increased knowledge

Twenty‐two of the 91 studies using DAs discussed the rates of surgery *versus* alternative therapies such as active surveillance. Some studies (n = 7) noticed an increase in surgical rates, particularly when tied to an increase in knowledge and when dealing with minority populations.^23^, ^24^, ^25^ The majority (n = 12) however noted a decrease in chosen surgical rates compared to more conservative treatment methods. This decrease in surgery was mirrored in the cross‐sectional studies. Where there was an increase in knowledge, patients in some of the studies (n = 2) determined that their knowledge of alternative treatments was also bolstered. Such an increase in knowledge, often resulting in a lower threshold of surgical pain expectation, was also coupled to decreased intervention rates. Other studies have noted an increase in physician–patient trust (n = 3), even if there was a decrease in the number of surgical interventions.

### 3.2 Decisional conflict

In 86% of the publications, decisional conflict was noted to decrease (n = 25) in intervention arms when compared to controls, with only two studies recording no difference between the control and study arms. This decrease was paralleled by a similar increase noted in decisional satisfaction (n = 9) when decisional conflict decreased. Four studies recorded decision quality, defined as the ability to make decisions aligned with the best information and patient values.^26^ Nearly all papers (n = 3) saw an increase in the quality of decision made. In only one study was there an increase in decisional conflict in the intervention arm using SDM. Similarly, where recorded, decisional anxiety was seen to decrease (n = 6). Decisional regret was also observed to decrease in the 5 studies using DAs.

### 3.3 Time spent and SDM preferred

Six papers recorded how much time was spent with SDM models (Figure 3). It was noted that time was seen to increase in three, decrease in two, and not change in one. Time was seen to increase on average by 10–30 minutes.

In each of these papers, it was also noted that there was an overwhelming support for SDM, recorded as SDM preferred over the usual treatment regimens, despite increased time spent in decision making. Of the studies that compared rates of SDM preference (5 of the 9), the increase in preference for SDM was high (range of 13% to 35%), whereas only a few (3 of 9) noted no difference in preference, with one noting a decrease in SDM preference.

### 3.4 Differences between DA types

**FIGURE 4:**
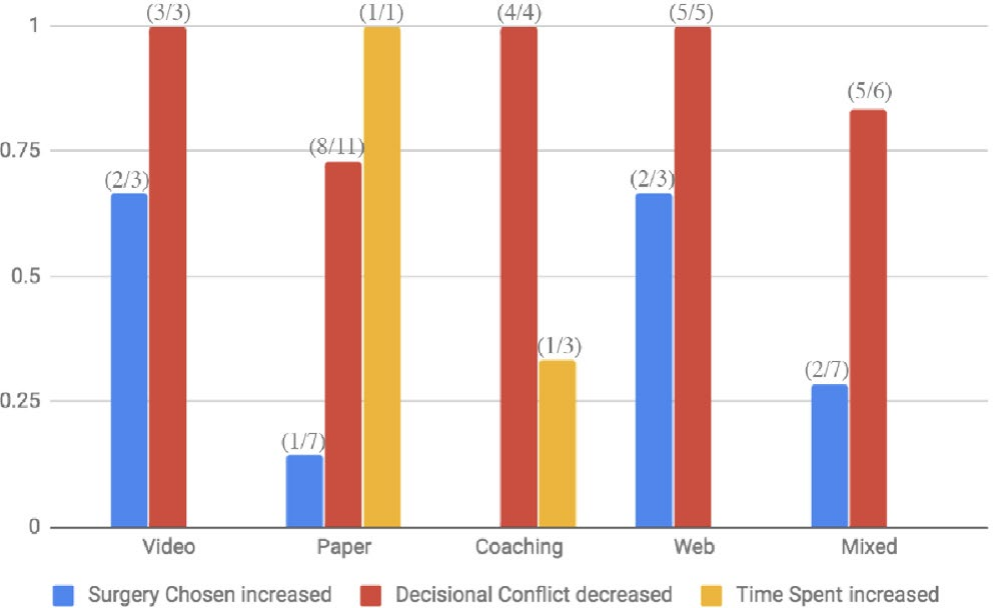
DA type with associated primary outcomes: surgery increased (blue), decisional conflict decreased (red), and time spent increased (yellow) using SDM

Several trends emerged when comparing the DAs with each other (Figure 4). Within the video intervention DA group (N = 9), rate of opting for surgery increased (n = 2) while decisional conflict decreased in each study that recorded it (n = 3). Among the paper‐based DAs (booklets, pamphlets; N = 30), the option of surgery compared to the non‐surgical intervention increased 14% of the time (n = 4) in the studies where it was recorded (N = 7); decisional conflict decreased 70% of the time (n = 7), and time spent in consult increased in all the instances it was recorded (n = 1). In coaching studies (N = 17), surgery did not increase in the papers that recorded the variable (N = 2), decisional conflict decreased in every instance (n = 5), and time spent discussing surgical options increased in 50% of the studies (n = 2).

Web‐based DAs (N = 19) increased surgery in 67% of those that recorded it (n = 3), decreased decisional conflict 100% of the time (n = 4), and showed no difference in time spent (n = 1) under SDM. Mixed media (N = 11) increased surgery in two of the instances (n = 7), decreased decisional conflict in 83% of the measures (n = 6), and decreased time spent in the single measure of the variable. Mobile apps and object‐based aids did not include enough studies that recorded the associated variables.

### 3.5 Narrative characteristics

Of the studies that did not specifically use a DA (n = 55) but still explored SDM in surgical disciplines, whether through qualitative surveys of departments or patient interviewing, trends emerged in the patients’ perception and expectation for particular care. While a large majority of the studies were focused on the comments from small samples (n = 28), a comparison between SDM and non‐SDM explorative studies was possible (Table 3). In studies that did not define their SDM protocol but rather explored the idea of using SDM in their select populations, there was a large degree of treatment agreement (94%), patient participation (83%), sense that the health care was simplified (54%), and less variability in treatment among institutions (24%). Among those that did not implement SDM in their surgical care, SDM was still highly preferred (84%). In non‐SDM surgical settings, there was limited value and role agreement (67% and 59%, respectively), and a significant amount of knowledge deficit was noted by patients (67%).

**TABLE 3:** Qualitative trends in non‐DA–specific surgical publications.^39^

	n = 27	Average (%)
SDM implemented		
Treatment agreement	2	0.945
Simpler	3	0.540
Patient participation	7	0.837
Treatment variety	2	0.244
No SDM		
SDM Desired	5	0.836
Value Agreement	2	0.67
Knowledge Deficit	2	0.485
Role Agreement	2	0.595
Complications Priority	2	0.365

## 4 DISCUSSION

Health care has moved towards patient‐centred approaches.^27^ With its widely studied use in screening, chronic conditions and cancer diagnosis, SDM is generally considered among the best current means to provide optimal treatment outcomes.^1^, ^2^, ^3^, ^4^ Many of the same driving effects of SDM were found in this review of surgical disciplines. We have observed that, generally, surgical rates and decisional conflict decreased, whereas knowledge obtained increased. Time spent with the patient, on the other hand, was inconclusive. This was true across all types of DAs used in SDM, though certain types (such as in‐person coaching) had a more consistent effect on specific outcomes such as decisional conflict. Narratively, a similar theme has been observed: patients prefer SDM, believing that their health care becomes more accessible, and treatment goals were aligned compared to those without SDM. These gains are reinforced by the uniform preference of patients undergoing surgery for SDM, a care that aligns with their values and preferences, and allows them to make the most informed decision in their care, especially when alternative treatment methods exist.

These results are both encouraging and consistent with prior literature.^28^, ^29^ Other reviews have also recorded a decrease in surgical rates, particularly as they relate to breast cancer interventions.^30^ This result is however only beneficial if the risks of not having surgery are outweighed by a patient's understanding and acceptance of both their current and future conditions.^31^ While our study did not deal with this aspect, they note that more conservative therapy appears to be preferred with SDM.

Of those studies that showed an increase in choosing surgical interventions, the effect was generally intended. Such for instance is a study in which the DA was tailored specifically to increase arthroplasty rate in racial minorities’ populations.^23^ This supports a well‐documented aspect of SDM research—racial minority and ethnic minority populations may benefit more than others from SDM paradigms.^24^, ^25^ Race/ethnicity of study participants was not reported, and therefore, any variation in the effect of the intervention across this demographic could not be ascertained. Other social determinants of health, like socio‐economic or education status, were also not recorded. As a result, the exact effect could not be quantified.

This is true of other patient characteristics too, such as the fact that many of the populations studied were older, with only one SDM protocol for paediatric populations. In those older populations, the knowledge of surgical procedures is largely considered lower, particularly for more advanced procedures.^32^ DAs may be a beneficial way to bridge this knowledge deficit, especially if SDM provides a reinforcing effect in minority populations. DAs would therefore provide a standardized way across the surgical disciplines to formalize care. Rather than vulnerable patients finding themselves voiceless in front of influential surgeons’ advice, SDM paradigms tend to equalize the patient–provider power balance within the therapeutic relationship, thereby, as observed, increasing physician trust.

In light of this effect of equalizing practitioners and patient, especially in minority populations, SDM has been lauded by the American Medical Association (AMA) as the future of medical practice.^17^ Unfortunately, similar calls have yet to come from surgical associations. As noted earlier, this may be due to the numerous limitations in surgery. Among them, time restrictions and generally shorter interactions are noted often as a barrier.^30^, ^31^ This study does not deny this concern, as indeed SDM occasionally does increase the time spent with patients—an important consideration before urgent or emergent surgery. In such instances, time is crucial and a trade‐off between life‐saving and option‐discussion focus needs to be carefully weighed, one case at a time.

A secondary reason for the limited use of SDM in surgery may be the perception that patients may not comprehend the complex and inter‐related health states associated with surgical complications.^17^, ^33^ Our hypothesis, however, is that this deficit can be lessened through the use of DAs, as seen in their general knowledge increase; that patients prefer understanding their procedure, as observed in their SDM partiality; and that the surgical knowledge deficit is not a reflection of the patient, but rather of the physician's time with them.^17^

No best practice or guideline on how to foster this mutual therapeutic relationship can be offered from this review. The DAs utilized showed practically equal ability to decrease decisional conflict, anxiety, and increase satisfaction and knowledge. While the sample size was small (n = 17), intervention rates were most decreased by using a face‐to‐face method, such as coaching. This may be due to the observed effect of patients feeling reassured through the close personal contact with their providers and developing a relationship with them, thereby choosing an alternative that is more fitting with their true preferences.^34^, ^35^ Other studies revealed that with more online medias like web‐based DAs, surgical rates increased. It is likely that these interventions facilitate a form of separation from direct physician contact, though they may be most accessible to certain surgical populations (such as younger patients). Further investigation into identifying best‐suited DAs for each surgical field and their impact on intervention rates would require larger sample sizes needed for multivariate analysis. This review has several limitations. The first is that many of the outcomes are patient‐reported. For example, decisional conflict was assessed largely using a standardized decisional conflict scale, but it does not include physician's input, focusing solely on patients’ inputs. Furthermore, the actual information and content of the DAs in the studies were not investigated. DAs that were not registered with the International Patient Decision Aids Standards (IPADS) were not separated from others that had registered.^36^ Our study also does not investigate comparative care regimes that define controls differently for the same procedure. This limitation is further exacerbated by the fact that the outcomes were often measured by author‐defined means, rather than using validated scales, such as the decisional conflict or the decisional regret scale.^36^, ^37^ This may be intentional, given the lack of rigor and standard of using SDM outcomes s in surgical disciplines.^37^

Finally, this study notes the incidence of SDM in various surgeries but cannot describe their varied use in any one surgery, as well as the outcomes of surgeries (such as less complications or infections). This is partly due to the limited spectrum of subspecialties. Most SDM studies within surgery were conducted in plastics, followed by general surgery and orthopaedics. Therefore, any extrapolations on other surgical specialties need to be guarded. This is also due to their varied use and definitions of SDM, even within the same discipline, as well as small sample sizes of each.

Despite these limitations, the implications for clinical practice are significant. If SDM can provide timely, appropriate care management for patients that coincides with their own values, then the possibility of undesired outcomes may be decreased. In particular, this research suggests that decisional regret will go down, knowledge regarding treatment modalities, and that a better therapeutic relationship would develop. Moreover, from the narrative review, patients appear to be preferring SDM, even in disciplines like surgery.

## 5 CONCLUSION

SDM is advocated for providing the utmost efficacious, reliable and patient‐focused care. This systematic review shows that the same principles guiding SDM in general also ground SDM in surgical settings. These findings can encourage the further application of SDM in surgery, especially for particular contexts such as elective procedures or in instances of equipoise where SDM is an extension of true informed consent.^35^, ^38^ Further studies would need to explore the types of DAs in their respective surgical fields, as well as the reasons for the wide variability in SDM penetration across surgical specialties. The potential unique application of surgical SDM for minority populations such as children, the elderly, and ethnic and racial minorities also warrants further study.

## AUTHORS’ CONTRIBUTIONS

Kacper Niburski involved in literature review and data analysis, and wrote, reviewed and edited the manuscript. Sadaf Mohtashami involved in literature review. Elena Guadagno designed the search strategy and reviewed and edited the manuscript. Dan Poenaru is a primary investigator, designed the concept, and reviewed and edited the manuscript.

## Data Availability

The authors confirm that the data supporting the findings of this study are available within the article [and/or] its supplementary materials.
